# Comparison of the Tomographic, Clinical Presurgical, and Intrasurgical Assessments of Furcation Involvement in the Lower Molars

**DOI:** 10.7759/cureus.80434

**Published:** 2025-03-11

**Authors:** Eliane Porto Barboza, Diogo Rodrigues, Caroline Montez, Rodrigo L Petersen, Beatriz Panariello

**Affiliations:** 1 School of Dental Medicine, Lake Erie College of Osteopathic Medicine, Bradenton, USA; 2 Periodontics, National Institute of Dental Science (INCO 25), Niteroi, BRA; 3 School of Dentistry, Augusto Motta University Centre (UNISUAM), Rio de Janeiro, BRA; 4 Private Practice, Petersen Imaging Diagnostic Clinic, Niteroi, BRA

**Keywords:** class ii furcation involvement, cone-beam computered tomography (cbct), cross-sectional, furcation involvement, periodontitis

## Abstract

This cross-sectional study compared cone-beam computed tomography (CBCT) measurements with clinical presurgical and intrasurgical measurements in the lower molars with furcation involvements (FIs). The sample consisted of 22 lower molars diagnosed with FI. The presurgical clinical examination included assessments of the probing depth, clinical attachment level, and horizontal bone loss (H-BL). Tomographic and intrasurgical measurements were taken to evaluate the vertical bone loss (V-BL), the distance of the cementoenamel junction to the base of the bone defect (CEJ-B), the horizontal bone loss (H-BL), root trunk length (RT), and furcation entrance width (FEW). Statistical analysis was performed, and values with p < 0.05 were considered statistically significant. The study of horizontal bone involvement showed a high level of agreement between presurgical, intrasurgical, and tomographic assessments. Strong correlations were found between intrasurgical and CBCT measurements for H-BL and furcation entrance width. Regarding the intrasurgical evaluation, class I FI was more prevalent, while class II FI was more frequently identified in CBCT evaluations. H-BL was significantly greater in class II FI compared to class I lesions. Moreover, the V-BL was greater in subclass B FI than in subclass A lesions. We found a strong correlation between horizontal furcation involvement measurements from CBCT and intrasurgical exams. CBCT was more accurate in assessing furcation morphology and vertical bone defects, making it a valuable complementary tool for decision-making in the surgical management of furcation involvements.

## Introduction

The treatment for preserving teeth with furcation involvement (FI) represents one of the biggest challenges in periodontal therapy [1]. The complex morphology of furcation areas predisposes them to biofilm accumulation, contributing to the ongoing destruction of periodontal tissues [2,3].

Understanding the anatomy of the root trunk and pre-furcation area, along with the width and length of the furcation area, provides crucial insights into the progression, prevention, and treatment of FIs [2]. The diagnosis of FI has traditionally relied on probing depth, clinical attachment level, furcation entrance probing, bitewing radiographs, and periapical radiographs [1]. One major limitation of conventional radiographic techniques is their ability to represent a three-dimensional structure in a two-dimensional image [4-7]. To overcome this, cone-beam computed tomography (CBCT) has increasingly been incorporated into routine dental assessments [5-7]. CBCT offers the advantage of high-resolution, three-dimensional imaging with lower radiation exposure and cost compared to conventional helical computed tomography, allowing for accurate assessment of the furcation defect's size, shape, and severity, which is often difficult to visualize with traditional 2D radiographs fully [6,7]. This detailed information is crucial for proper diagnosis and treatment planning in periodontal cases involving FI.

The accuracy of CBCT for diagnosing periodontal and furcation lesions has been well-established in both *in vitro* and clinical studies [8-16]. In addition, CBCT enables a detailed evaluation of the supporting bone, the proximity of each root to adjacent dental structures, and the presence of root fusions [6,7,11]. Human studies have well-documented the accuracy of CBCT in evaluating the furcation region of upper molars [8-10,12]. One study highlighted that the most significant discrepancy between clinical assessments and tomographic measurements occurred in the vestibular region of lower molars, which is typically the most accessible area for probing [16,17]. Therefore, the present study aimed to compare measurements obtained through CBCT with pre-surgical and intra-surgical clinical measurements in the furcation areas of mandibular molars. 

By comparing presurgical and intrasurgical clinical measurements with CBCT, our study provides valuable insights into the clinical relevance and practical application of CBCT in evaluating FI, particularly in mandibular molars. The specific objective of the study was to compare the pre-surgical clinical measurements, which typically include probing depth, clinical attachment level, and horizontal bone loss, with both intra-surgical measurements and tomographic measurements obtained through CBCT. By doing so, the study aimed to determine the level of agreement and correlation between these different assessment methods, focusing on the furcation entrance width, the vertical and horizontal bone loss, and root trunk length. Furthermore, the study sought to explore the differences between clinical, intrasurgical, and CBCT evaluations regarding their ability to accurately classify the severity of FI, which can have significant implications for treatment planning. The ultimate goal of this research was to establish the potential of CBCT as a complementary tool in managing FI, potentially improving diagnostic accuracy and aiding in the decision-making process for surgical intervention.

In summary, this study aims to contribute to the ongoing discussion on the accuracy of CBCT and its potential advantages over traditional clinical methods in complex cases, ultimately improving diagnostic accuracy and treatment planning in periodontal care. The null hypothesis of this study is that there is no significant difference between CBCT measurements and clinical presurgical and intrasurgical measurements in assessing FI and related parameters in the lower molars. The alternative hypothesis is that there is a significant difference between CBCT measurements and clinical presurgical and intrasurgical measurements in assessing FI and related parameters in the lower molars.

## Materials and methods

This cross-sectional study was conducted at the Multidisciplinary Clinic of the Fluminense Federal University (FFU), School of Dental Medicine, Niterói, Brazil, with protocol approval from the FFU School of Medicine Ethics Committee (CEP/HUAP 840.007) and followed the Strengthening the Reposting of Observational Studies in Epidemiology (STROBE) guidelines. Participants aged 45 to 68 were recruited between January 2022 and May 2022. Informed consent was obtained from all participants. Inclusion criteria required a periodontitis diagnosis with lower first or second molars showing FI and no more than 2 mm gingival recession. Patients were excluded if they presented with teeth exhibiting furcation caries or restorations close to the bone crest, were pregnant, had systemic or uncontrolled chronic conditions, had motor disorders affecting oral hygiene, were smokers, had received head or neck radiotherapy within the past five years, or were on continuous bisphosphonate therapy.

A power analysis referencing the work of Qiao et al. [9], who evaluated molar furcation using clinical probing and CBCT, suggested that a sample size of 51 would yield 80% power to identify associations at a significance level of 0.05. For the present study, 22 FI sites were selected for analysis. Although this sample size is smaller than expected, the focus on furcation defects and the study's nature - incorporating clinical and intrasurgical measurements - required a smaller sample. In any case, the methodology of the present study was carefully designed to detect meaningful patterns, and the sample size is sufficient to detect medium to large effects (α = 0.05).

Therefore, 22 FIs in 15 mandibular molars (eight first molars and seven second molars) were analyzed in this study and were evaluated through both tomographic and clinical assessments conducted pre- and intrasurgically. Basic periodontal therapy, which included oral hygiene instruction, scaling and root planing of all teeth, and occlusal adjustment, was provided to all participants. Periodontal surgery was performed on those with at least one lower molar exhibiting FI, for which surgical intervention was deemed necessary. Before surgery, all participants underwent a CBCT scan (Prexion 3D Elite unit, San Mateo, CA, USA). Each participant received treatment tailored to the degree of FI diagnosed. Preoperative evaluations, including a complete blood count, coagulogram, and glucose levels, such as the hemoglobin A1C, were also conducted. Preoperative tests are necessary to assess the participants' overall health and determine their ability to undergo surgery safely. These tests help to prevent potential complications during surgery, ensuring that only those who are medically fit are included for the surgical procedures. The tomographic and clinical measurements were taken between June 2022 and April 2024.

Tomographic measurements (CBCT scans)

**Figure 1:**
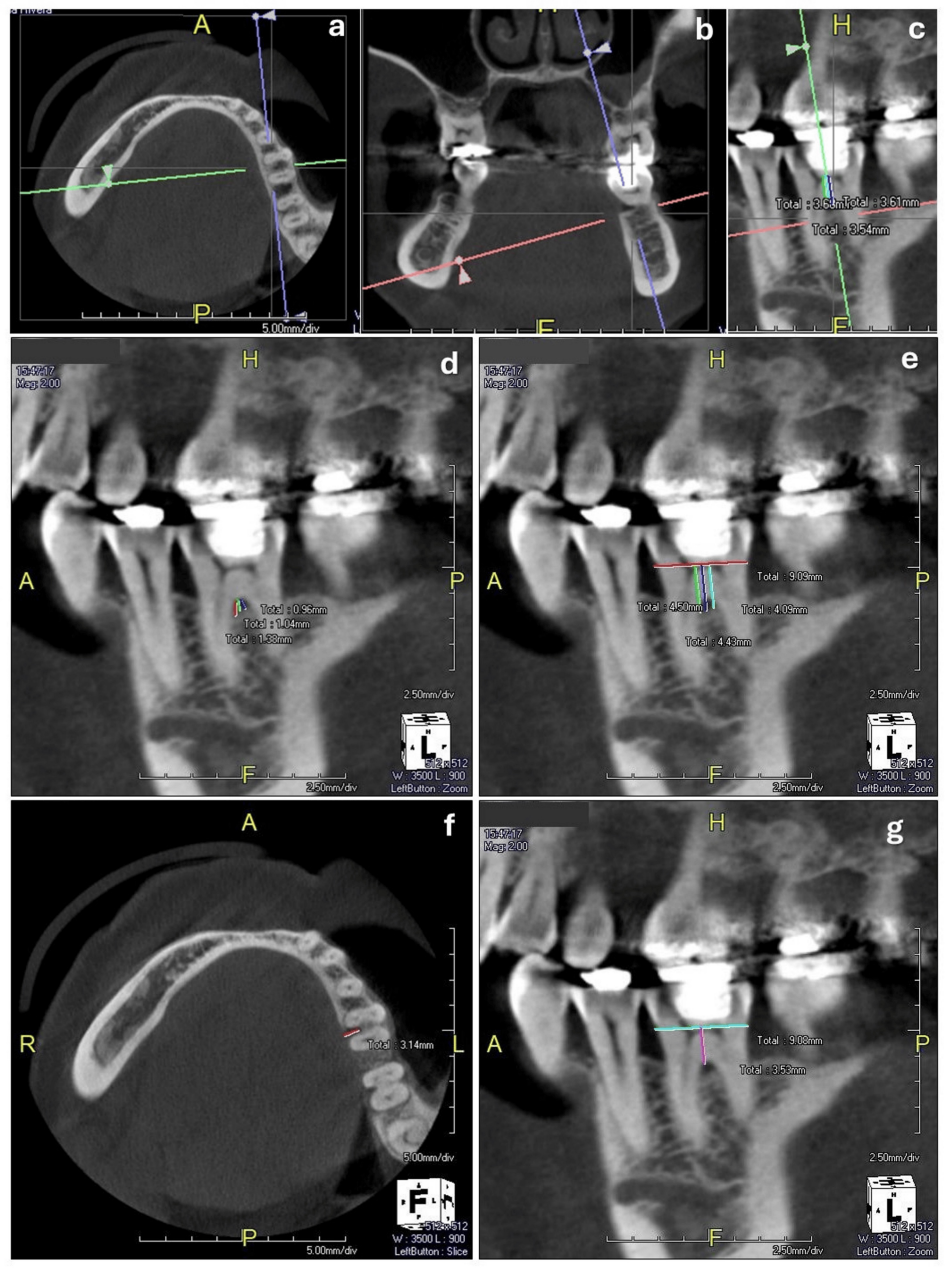
Tomographic measurements. a: Axial plane; b: Coronal plane; c: Sagittal plane; d: Vertical bone loss (V-BL) was measured from the entrance of the furcation to the base of the bone defect in the vertical direction; e: Vertical bone loss was also measured from the cementoenamel junction to the base of the bone defect (CEJ-B); f: Horizontal bone loss (H-BL) was measured from the distance between the outer surface of the root and the bone between the roots; g: The length of the root trunk (RT), buccal and lingual, was represented by the distance between the CEJ and the entrance of the furcation.

CBCT scans were performed at the Petersen Diagnostic Imaging Clinic (Niterói, Rio de Janeiro, Brazil). The exams were obtained using the Prexion 3D Elite unit, and the images were analyzed using the Prexion 3D Viewer software (Prexion Inc., San Mateo, CA, USA). The participants were positioned with their chin and head stabilized for the tomographic examination. The unit was set at 90 kVp and 4 mA. The field of view used was 5 x 5 mm, the focal size was 0.15 mm, and the voxel size was 0.11 mm. A radiologist blinded by the clinical examination performed the measurements (RLP). All captured images were displayed on a 32-inch flat panel screen (HP Development Co.) with a 1920 × 1080-pixel resolution. The three axes (X, Y, and Z) in the sagittal, coronal, and axial planes of the CBCT images were obtained. Based on the classification of Hamp et al. [18], the horizontal bone loss (H-BL) was measured from the distance between the root's outer surface and the bone between the roots in section Z. The following measurements were taken in section X. Vertical bone loss (V-BL) was measured from the entrance of the furcation to the base of the bone defect in the vertical direction, according to the classification by Tarnow and Fletcher [19]. Vertical bone loss was also measured from the cementoenamel junction to the base of the bone defect (CEJ-B). The length of the root trunk (RT), buccal and lingual, was represented by the distance between the CEJ and the entrance of the furcation (Figure 1).

The furcation entrance width (FEW) was measured at the buccal and lingual furcations. All measurements were performed three times, and the mean value was used to map the FI.

Presurgical and intrasurgical measurements

**Figure 2:**
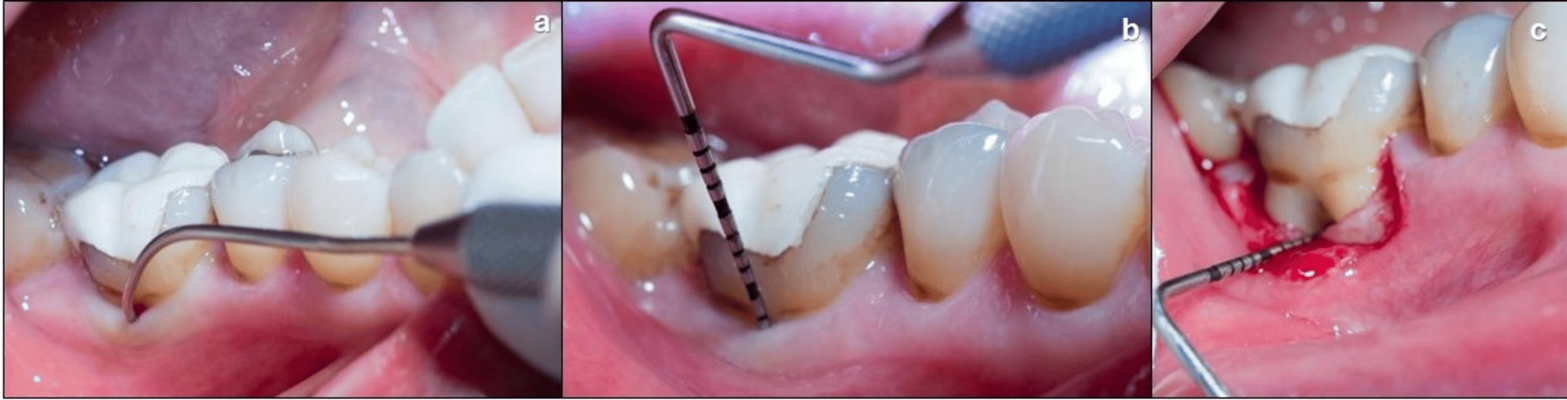
Clinical measurements. a: Presurgical horizontal measurement. b: Presurgical vertical measurement. c: Intrasurgical horizontal measurement.

The clinical presurgical examination of the molars was conducted by an experienced periodontist calibrated before the study and blinded to the results of the tomographic scans. Clinical parameters, such as probing depth (PD) at six sites (mesiobuccal, buccal, distobuccal, mesiolingual, lingual, and distolingual) and clinical attachment level (CAL), were measured using a periodontal probe (PCPUNC-15; Hu-Friedy, Chicago, IL, USA). Buccal and/or lingual FI was assessed with a Nabers probe (PQ2N; Hu-Friedy, Chicago, IL, USA) (Figure 2a, 2b). 

Measurements were recorded according to the classification systems proposed by Hamp et al. (1975) [18] and Tarnow and Fletcher (1984) [19]. The decision to use these classification systems is based on their long-standing acceptance and validation within the field. These systems are widely used in clinical practice and research, providing a consistent framework for evaluating FI. 

The intrasurgical examination of the molars was performed under local anesthesia by an experienced periodontist blinded to the results of the tomographic scans. The flaps were elevated, the bone defects were debrided, and the root surface was properly instrumented. Using a periodontal probe (PCPUNC-15; Chicago, IL, USA), the following measurements were performed: H-BL, V-BL, CEJ-B, RT, and FEW (Figure 2c). The flaps were sutured with 4-0 resorbable thread. The patients were instructed to rinse with 0.12% chlorhexidine gluconate twice daily for 14 days. Ibuprofen 400 mg every eight hours as needed was prescribed for pain relief as needed. The participants were re-evaluated within 14 days and taken to a maintenance program according to their needs.

Calibration and reproducibility of clinical and tomographic measurements

The reproducibility of continuous variables was assessed using the intraclass correlation coefficient (ICC), with values greater than 0.75 considered excellent. The reproducibility of categorical variables was evaluated using the Kappa coefficient, where values greater than 0.80 were considered strong. To ensure accuracy and reproducibility, all clinical measurements were performed by the same examiner (EPB). The intra-examiner Kappa coefficient for the degree of horizontal furcation between these two measurements was 0.801.

For the tomographic measurements, the same examiner conducted all measurements and repeated them 15 days later (RLP). The intra-examiner Kappa coefficient for the degree of furcation was 0.904. The intraclass correlation coefficient for the three measurements of each tomographic variable was excellent, with all variables showing a correlation greater than 0.895 (p<0.001).

Statistical analysis

Statistical analyses were performed using IBM SPSS Statistics for Windows, Version 23.0 (released 2015, IBM Corp., Armonk, NY). FI on each tooth was treated as a unit for all analyses. The normality of the data was assessed using the Shapiro-Wilk and Kolmogorov-Smirnov tests. Descriptive statistics were calculated for all continuous variables, including mean values and standard deviations. A total of 22 FI cases were analyzed. The Chi-square test (X²) was used to assess the association between the clinical presurgical, intrasurgical, and CT scan measurements of the horizontal FI and between intrasurgical and CT scan classifications of vertical FI. To compare the distribution of variables such as root trunk length (RT), horizontal bone loss (H-BL), vertical bone loss (V-BL), cementoenamel junction to bone (CEJ-B), and furcation entrance width (FEW) between the first and second molars, buccal and lingual furcation, and the degrees of horizontal and vertical FI in both tomographic and intrasurgical evaluations, the Mann-Whitney and Kruskal-Wallis tests were used. Spearman's correlation test was employed to examine the correlations between horizontal and vertical bone loss across clinical, intrasurgical, and CT analyses. A p-value of <0.05 was considered statistically significant.

## Results

A total of 22 FIs in 15 mandibular molars (eight first molars and seven second molars) were analyzed in this study. The analysis was divided into two FI groups: 10 buccal and 12 lingual furcations. Tomographic measurements of the root trunk were conducted regardless of the presence of furcation lesions, resulting in 30 root trunk measurements (15 buccal and 15 lingual). The average root trunk measurements for the first molars were 3.51 ± 0.77 mm on the buccal side and 3.34 ± 0.73 mm on the lingual side. For second molars, the average measurements were 3.58 ± 0.85 mm on the buccal side and 3.24 ± 0.44 mm on the lingual side. The clinical presurgical examination assessed horizontal bone loss, revealing an average of 4.00 ± 1.90 mm, while the vertical clinical attachment level measurement averaged 4.24 ± 1.32 mm.

Comparison of tomographic, clinical presurgical, and intrasurgical examinations

The association between the different methods of evaluating FI (chi-square test, p < 0.05) and the kappa level of agreement are presented in Table 1. 

**Table 1:** Association between the different methods of evaluation of furcation involvement. K: Kappa concordance test, X2: Chi-square association test. * denotes statistical significance (p < 0.05).

Degree of furcation involvement	K	X^2^	P-value
Horizontal	-	-	-
Clinical presurgical vs. intrasurgical	0.808	70.819	0.000*
Tomographic vs. clinical presurgical	0.902	78.262	0.000*
Tomographic vs. intrasurgical	0.692	79.744	0.000*
Vertical	-	-	-
Tomographic vs. intrasurgical	0.353	4.714	0.003*

A high kappa agreement (>0.9) was observed for the degree of horizontal FI between the clinical presurgical and intrasurgical examinations and between the clinical presurgical and tomographic examinations. However, the agreement between tomographic and intrasurgical examinations for the degree of horizontal involvement was moderate, while for vertical involvement, it was low.

The mean measurements for the various parameters evaluated during surgery and on tomographic examinations are presented in Table 2.

**Table 2:** Intrasurgical and tomographic measurements. Mann-Whitney U test, * denotes statistical significance (p < 0.05). Abbreviations: SD: standard deviation, CEJ-B: vertical bone loss from the cementoenamel junction (CEJ) to the base of the bone defect, V-BL: vertical bone loss from the furcation entrance to the bone base, RT: root trunk, H-BL: horizontal bone loss, FEW: furcation entrance width

Intrasurgical							
Measurements	General (n = 22) Mean ± SD	First molar Mean ± SD	Second molar Mean ± SD	P-value	Buccal (n = 10) Mean ± SD	Lingual (n = 12) Mean ± SD	P-value
CEJ-B	5.92±1.17	5.82±1.23	6.01±1.16	0.699	6.32±1.15	5.58±1.12	0.254
V-BL	2.54±0.80	2.45±0.93	2.63±0.67	0.652	2.20±0.91	2.83±0.57	0.159
RT	2.54±0.85	2.27±1.01	2.81±0.60	0.332	2.50±0.97	2.58±0.79	0.821
H-BL	4.36±2.03	4.45±2.38	4.27±1.73	0.898	3.20±1.75	5.33±1.77	0.014*
FEW	1.90±0.61	1.90±0.83	1.90±0.30	0.949	1.70±0.48	2.08±0.67	0.228
Tomographic							
Measurements	General (n = 22) Mean ± SD	First molar Mean ± SD	Second molar Mean ± SD	P-value	Buccal (n = 10) Mean ± SD	Lingual (n = 12) Mean ± SD	P-value
CEJ-B	5.17±1.21	4.91±1.05	5.43±1.36	0.606	4.49±1.01	5.74±1.10	0.021*
V-BL	2.75±1.20	2.68±1.37	2.82±1.07	0.562	2.67±1.27	2.81±1.19	0.674
RT	3.47±0.70	3.56±0.65	3.38±0.76	0.401	3.62±0.86	3.35±0.52	0.582
H-BL	3.79±1.68	3.68±1.65	3.90±1.77	0.652	3.14±1.77	4.33±1.45	0.123
FEW	1.85±0.56	2.20±0.46	1.50±0.43	0.004*	1.93±0.64	1.79±0.51	0.418

Only horizontal bone loss observed during the intrasurgical examination showed a significant difference between the buccal and lingual furcations. In the tomographic examination, the vertical distance between the CEJ and bone (CEJ-B) was significantly smaller in the buccal furcation compared to the lingual furcation. In addition, the furcation entrance width was considerably smaller in the second molar than in the first molar.

Table 3 compares the degree of FI (class I, II, or III) and the parameters evaluated.

**Table 3:** Comparison of tomographic and intra-surgical horizontal bone loss at each FI classification. Kruskal-Wallis test, * denotes statistical significance (p < 0.05). Abbreviations: CEJ-B: vertical bone loss from the cementoenamel junction (CEJ) to the base of the bone defect, V-BL: vertical bone loss from the furcation entrance to the bone base, RT: root trunk, H-BL: horizontal bone loss, FEW: furcation entrance width

Intrasurgical					Tomographic			
Measurements	Class I (n = 11)	Class II (n = 9)	Class III (n = 2)	P-value	Class I (n = 7)	Class II ( n = 13)	Class III (n = 2)	P-value
CEJ-B	5.74±1.25	6.15±1.24	5.82±0.24	0.998	5.24±1.17	5.09±1.22	5.49±2.11	0.998
V-BL	2.45±0.82	2.66±0.86	2.50±0.70	0.485	2.30±0.40	2.77±1.41	4.14±0.38	0.266
RT	2.45±1.03	2.55±0.72	2.59±0.82	0.554	3.87±0.57	3.34±0.72	2.94±0.02	0.000*
H-BL	3.27±2.24	5.44±0.88	5.50±2.12	0.139	2.06±0.67	4.33±1.24	6.35±0.06	0.126
FEW	1.54±0.52	2.33±0.50	1.83±0.07	0.866	1.98±0.62	1.79±0.59	1.83±0.07	0.771

Only in the tomographic measurements was the height of the root trunk significantly different in the different classes of FI. The RT was lower in class II than in class I. Class III had the lowest RT; however, only two furcations with that class were evaluated. In the intra-surgical evaluation, class I was more prevalent, while class II was more prevalent in tomographic evaluation.

The comparison of vertical FI (subclass A, B, or C) with the evaluated parameters revealed that class A was the most prevalent in both the tomographic and intra-surgical clinical examinations. On CT scans, the distance from the furcation entrance to the bone base and the horizontal bone loss were significantly greater in subclass B compared to subclass A lesions. These findings are shown in Table 4.

**Table 4:** Comparison of intra-surgical and tomographic measurements for each subclass of furcation involvement. Mann-Whitney U test, * denotes statistical significance (p < 0.05). Abbreviations: CEJ-B: vertical bone loss from the cementoenamel junction (CEJ) to the base of the bone defect, V-BL: vertical bone loss from the furcation entrance to the bone base, RT: root trunk, H-BL: horizontal bone loss, FEW: furcation entrance width

Intrasurgical					Tomographic			
Measurements	A (n = 15)	B (n = 7)	C (n = 0)	P-value	A (n = 20)	B (n = 2)	C (n = 0)	P-value
CEJ-B	4.97±0.85	5.62±1.77	-	0.298	5.89±1.23	6.16±0.23	-	0.701
V-BL	2.05±0.42	4.26±0.86	-	0.000*	2.50±0.82	2.34±0.29	-	0.485
RT	3.68±0.70	3.03±0.48	-	0.066	2.50±0.88	2.63±0.16	-	0.554
H-BL	3.07±1.21	5.34±1.52	-	0.007*	4.15±2.00	6.50±0.70	-	0.139
FEW	1.83±0.57	1.91±0.59	-	0.630	1.90±0.64	2.07±0.19	-	0.866

A strong correlation between intrasurgical and CT scan measurements for horizontal bone loss is shown in Table 5. 

**Table 5:** Spearman and intraclass correlation analysis between tomographic and intrasurgical measurements. Spearman's test; * denotes statistical significance (p < 0.05). Abbreviations: Rho: Spearman's test, CEJ-B: vertical bone loss from the cementoenamel junction (CEJ) to the base of the bone defect, V-BL: vertical bone loss from the furcation entrance to the bone base, RT: root trunk, H-BL: horizontal bone loss, FEW: furcation entrance width. Intraclass correlation test, * denotes statistical significance (p < 0.05)

Measurements	Rho	P-value	Intra-class correlation	P-value
CEJ-B	0.249	0.264	0.152	0.244
V-BL	0.058	0.797	0.006	0.488
RT	0.341	0.121	0.227	0.149
H-BL	0.811	0.000*	0.338	0.057
FEW	0.346	0.115	0.757	0.000*

ICC analysis revealed that significant similarity between the two examinations was found solely for the furcation entrance width measurement.

## Discussion

This investigation compared the tomographic, clinical presurgical, and intrasurgical assessments of FI in lower molars. We found high agreement in horizontal FI between clinical presurgical and intrasurgical exams and between clinical presurgical and tomographic assessments. However, the agreement between CBCT and intrasurgical exams for horizontal FI was moderate and weak for vertical furcation subclassification, likely due to differences in measurement precision. The periodontal probe records measurements at 1 mm intervals, whereas CBCT provides much higher precision, recording to 0.01 mm [9,14,15,20]. Consequently, in cases near the borderline between class I and II or subclass A and B, discrepancies may arise between intrasurgical and tomographic measurements due to the differing levels of precision in the two diagnostic tools.

This study used the classification of horizontal FI by Hamp et al. (1975) [18]. By contrast, other studies involving lower molars have employed Glickman's classification (1958) [21], which defines class I as an "early or incipient lesion" but lacks specific numerical values to quantify horizontal attachment loss [14,21,22]. Hamp's classification may struggle to distinguish between class I and II, as both rely on the same reference point (less than or greater than 3 mm). To address this, a new FI classification system was proposed in 2018, incorporating the relationship between horizontal bone loss and the presence or absence of gingival recession [22]. However, the furcation area presents complex anatomical morphology that can be difficult to assess clinically, regardless of the classification system used. Therefore, complementary imaging, such as CBCT, should be considered essential for the accurate evaluation and diagnosis of FI.

While clinical assessment through probing and two-dimensional periapical radiography are the most used diagnostic methods for evaluating FI, both have notable limitations [6,7]. Probing FI is influenced by factors such as the clinician's knowledge of anatomy, probing technique, furcation entry width, and root trunk length [6,7]. In addition, periapical radiography tends to underestimate the lesion because it represents a three-dimensional structure in two dimensions and lacks the numerical precision needed to accurately assess horizontal and vertical bone loss [6-8]. By contrast, CBCT has been recognized as a valuable diagnostic tool for periodontal evaluations [7,8,17].

Several studies have confirmed the utility of CBCT in assessing FI in maxillary molars, where anatomical access to the furcation is more challenging for clinical probing [8-10,12]. Results in lower molars are inconsistent, with some studies showing conflicting findings. One study found the buccal surface of mandibular molars had the lowest agreement (36.6%) between clinical and CBCT measurements despite being the most accessible for direct examination [16]. Another study reported an 84% agreement between CBCT and presurgical assessments for FI on the buccal aspect of lower molars [20]. In the present study, the agreement for horizontal FI was strong, with kappa values of 0.902 between clinical and tomographic measurements and 0.808 between clinical and intra-surgical measurements. Clinical and tomographic evaluations showed 100% agreement with the intrasurgical assessment for class III FI, which aligns with existing literature [9,14].

Our results show that CBCT images accurately evaluated and classified periodontal tissue loss, aligning well with intrasurgical findings. Previous studies have also concluded that CBCT is significantly more effective than intraoral radiography in diagnosing class I and II FIs [23-25], with one study highlighting the advantages of time and cost savings of using CBCT for diagnostic planning before invasive treatment of periodontitis in molars [25]. Class II FI was more prevalent in the present study's clinical pre-surgical and tomographic evaluations, while class I was more commonly observed in the intra-surgical measurements. This finding supports other studies suggesting that tomography provides more accurate results than periodontal probing [9,14,15]. Class I and II FIs are typically suitable for guided tissue regeneration, while class III FIs usually require resective procedures or extraction [26-29].

An essential goal of regenerative therapy is the predictability of clinical furcation defect closure following periodontal regeneration [30,31]. The morphology of the furcation and the treatment plan play a significant role in determining the prognosis for class II furcation closure. Factors such as horizontal and vertical bone loss, probing depth, root trunk length, root divergence, and concavities are linked to successful furcation defect closure [30]. Generally, less severe defects are associated with a higher likelihood of achieving complete clinical furcation closure [30,31].

In this context, CBCT's accuracy is crucial for early diagnosis, treatment planning, and prognosis in periodontal surgery. The vertical component of FI has been associated with an increased risk of tooth loss during periodontal therapy [32]. In the present study, the mean difference between CBCT (2.75 mm) and intrasurgical examination (2.54 mm) was 0.21 mm, which aligns with previous studies reporting differences of less than 0.36 mm between these two methods [9,14].

All measurements in this study showed a difference of less than 1 mm between CBCT and intrasurgical probing, further supporting the notion that CBCT provides results comparable to intrasurgical measurements, which are considered the gold standard in diagnosing FI.

Evaluating the pre-furcation area and root trunk is crucial for predicting the prognosis of periodontal disease, as long root trunks present more significant challenges regarding operative access [33]. Root trunk length was statistically significant across different FI degrees when assessing horizontal FI. While previous studies report a longer root trunk on the lingual side, our study found it to be longer on the buccal side [34,35]. For instance, Perminio et al. (2021) used microtomography to evaluate root trunk length, reporting averages of 2.49 mm on the buccal and 3.18 mm on the lingual in mandibular first molars [35]. In a separate study assessing the RT of 96 extracted lower first molars [2], the clinical measurements revealed mean root thicknesses of 3.07 mm (buccal) and 3.54 mm (lingual). By contrast, the present study found tomographic averages of 3.51 mm (buccal) and 3.34 mm (lingual) for mandibular first molars. These differences in measurements could be attributed to variations in the methodologies employed.

In this study, horizontal and vertical bone loss was significantly greater in subclass B FI compared to subclass A lesions. Subclass B defects were associated with a mean horizontal bone loss (H-BL) of 5.34 ± 1.52 mm on CBCT and 6.50 ± 0.70 mm on intrasurgical probing. In addition, the mean root trunk length measured on CBCT was 0.65 mm shorter in subclass B. These findings suggest that teeth with larger root trunks tend to have a poorer prognosis when FI is present. This is likely due to the increased difficulty in accessing and instrumenting the furcation area, which complicates effective periodontal treatment and regeneration [2].

Furthermore, we observed a strong correlation between intrasurgical and tomographic measurements of furcation width. This result is consistent with the study by Padmanabhan et al. (2017), which reported a mean furcation width of 1.87 mm on CBCT and 1.84 mm intrasurgically [14]. Similar measurements were obtained in our study, with values of 1.85 mm on CBCT and 1.90 mm in intrasurgical evaluations. However, these results contrast with those of Bower (1979), who found that 81% of upper and lower furcation entries measured only 1 mm in width [36]. This discrepancy is likely due to the enhanced precision of modern imaging techniques, which allow for more accurate measurements of furcation morphology.

Furcation instrumentation continues to pose a significant challenge for periodontists due to the considerable variability in furcation entry, root morphology, and bone defect architecture [37]. Understanding the width of the furcation entrance is crucial for selecting the appropriate surgical instruments, such as manual curettes (0.75-1.10 mm) or ultrasound tips (0.50 mm) for treatment [30,37]. In addition, root divergence in FI has been identified as a key predictor of successful furcation closure following regenerative therapy [30]. Therefore, accurately evaluating these parameters is essential for optimizing treatment outcomes. In our study, the correlation between intra-surgical and tomographic assessments of horizontal bone loss was strong, highlighting the value of CBCT as a complementary tool for evaluating FI morphology. This is especially important when considering more invasive treatments, such as regenerative procedures, particularly in class II FI cases [9,11,14,15].

CBCT image quality is influenced by several acquisition parameters, including milliamperage, kilovoltage, and voxel size [38]. Smaller voxel sizes and narrower fields of view (FOV) are associated with increased sensitivity and diagnostic accuracy for detecting periodontal defects, as shown in various in vitro studies [24,39]. One study comparing different voxel resolutions and FOV sizes identified a voxel size of 0.15 mm as the optimal threshold for detecting periodontal defects [40]. The present study used a 5 x 5 mm FOV and a 0.11 mm voxel size, providing even higher resolution than the recommended cut-off. Previous studies on FI in humans utilized larger voxel sizes than those recommended for periodontal defects, which may have influenced their findings [13,14].

CBCT offers a significant advantage in cases requiring invasive periodontal treatments for FI, as its radiation exposure is seven times lower than medical helical tomography and only 1.8 times higher than panoramic radiography [9,11,14]. The close agreement between CBCT and intrasurgical probing underscores CBCT's value as a presurgical tool for accurately assessing vertical bone defects. A systematic review of biomaterials for FI treatment found that regenerative therapies can reduce furcation defects from class II to class I [29]. Understanding the lesion's morphology via CBCT aids in selecting the most appropriate biomaterial and supports better surgical planning. In addition, CBCT outperforms clinical probing for monitoring the furcation area after regenerative therapy [15].

This study underscores the value of high-quality CBCT imaging in accurately assessing FI. The strong correlation between CBCT and intra-surgical probing highlights the potential of CBCT as a reliable pre-surgical tool. In addition, the study supports previous findings on the advantages of using smaller voxel sizes and narrower FOV for enhanced diagnostic accuracy. Despite the relatively small sample size of 22 lower molars, which may limit the generalizability of the findings to larger or more diverse populations, the rigorous statistical methods employed in this study ensure that meaningful conclusions can still be drawn. The strength of this research lies in its comparison of high-resolution tomographic imaging with both pre-surgical and intra-surgical clinical measurements, providing valuable insights into the role of CBCT in managing FI. Future studies with larger sample sizes could further validate these findings and expand their applicability across different demographic groups.

## Conclusions

Within the limitations of this study, we conclude that there is a strong correlation between the measurement of horizontal FI in tomographic and intra-surgical exams. By contrast, clinical presurgical probing alone was insufficient for an accurate diagnosis of FI. CBCT proved to be highly precise in assessing the morphology of the furcation area and vertical bone defects, making it a valuable complementary tool in planning the surgical treatment for FI.
